# Dynamics of tumor evolution after Gamma Knife radiosurgery for sporadic vestibular schwannoma: Defining volumetric patterns characterizing individual trajectory

**DOI:** 10.1093/neuonc/noae187

**Published:** 2024-09-16

**Authors:** Anne Balossier, Madalina Olteanu, Christine Delsanti, Lucas Troude, Jean-Marc Thomassin, Pierre-Hugues Roche, Marie Chavent, Jean Régis

**Affiliations:** Aix Marseille University, INSERM, INS, Inst Neurosci Syst, Marseille, France; Functional and Stereotactic Neurosurgery, AP-HM, Timone Hospital, Marseille, France; CEREMADE, UMR 7534, Université Paris Dauphine PSL, Paris, France; Functional and Stereotactic Neurosurgery, AP-HM, Timone Hospital, Marseille, France; Department of Neurosurgery, AP-HM, North University Hospital, Marseille, France; Department of Head and Neck Surgery, AP-HM, Timone Hospital, Marseille, France; Department of Neurosurgery, AP-HM, North University Hospital, Marseille, France; UMR5251, INRIA, Université de Bordeaux, Talence, France; Aix Marseille University, INSERM, INS, Inst Neurosci Syst, Marseille, France; Functional and Stereotactic Neurosurgery, AP-HM, Timone Hospital, Marseille, France

**Keywords:** failure, patterns, radiosurgery, vestibular schwannoma, volumetry

## Abstract

**Background:**

The definition of tumor control and treatment failure after Gamma Knife radiosurgery (GKRS) for vestibular schwannoma (VS) is still debated. The lack of knowledge on the dynamics of tumor evolution can lead to misinterpretation and subsequent inappropriate second treatment. The aim of this study was to evaluate the post-GKRS dynamics of the evolution of tumor volume and characterize volumetric patterns.

**Methods:**

We included patients with sporadic VS treated by GKRS with an MRI follow-up of a minimum of 3 years. A clustering was performed in 2 steps: Definition of the patterns of evolution based on a subset of patients with the most comprehensive follow-up, and then the assignment of the remaining patients on a best-fit basis. The minimum length of follow-up was assessed by measuring the consistency of the clusters over time (adjusted rand index and normalized mutual information). An analysis of the discriminant variables was finally performed.

**Results:**

A total of 1607 patients were included (median follow-up: 67 months). Five patterns were defined with 1 pattern gathering almost all cases of treatment failure. The clustering at 5 years afforded the highest consistency with long-term follow-up. Discriminant variables for clusters were as follows: sex, initial symptoms, delay of diagnosis, Koos grading, fundus invasion, and number of isocenters.

**Conclusions:**

The definition of these robust distinct patterns is likely to help the physicians tremendously to distinguish tumor control from potential failure. We advocate for no retreatment decision before 5 years post-GKRS. Further investigations are required to decide if the dynamics of evolution can be predicted at GKRS on an individual basis.

**Key Points**

- The definition of failure in terms of delay of follow-up and dynamics of evolution of the tumor volume is still a matter of debate.
- We advocate for a minimum delay of 5 years post-GKRS before concluding as a failure.

**Importance of the Study**

The absence of knowledge of the specific patterns of evolution after SRS can lead to a misinterpretation and inappropriate second treatment. The definition of these robust distinct patterns is likely to help the physicians tremendously to distinguish successful tumor control from potential failure in the longer term.

Stereotactic radiosurgery (SRS) has emerged in the last 30 years as one of the main treatment options for small to medium vestibular schwannoma (VS) due to its high tumor control rate, low morbidity, and higher postoperative quality of life compared to microsurgical resection.^1^ In most series, tumor control is reported with a wide range between 90% and 99%.^2^ Yet although more than 133 000 patients have been treated worldwide only considering Gamma Knife radiosurgery (GKRS), the definition of tumor control and more precisely of treatment failure still varies across physicians, considering both the timing post-SRS and volumetric criteria of progression. Few authors have attempted to evaluate the morphological changes occurring after SRS in order to define the cure-failure parameters, but this remains a matter of debate.^3^ The absence of knowledge of the specific patterns of evolution after SRS can lead to misinterpretation and inappropriate second treatment with potential consequences for the patient. Moreover, the debate on the criteria of failure is often used as an argument against SRS. The aim of this study was to evaluate, in a historical cohort of patients treated by GKRS for VS, the dynamics of tumor evolution, characterize volumetric patterns associated with specific trajectories, and define the minimal delay of follow-up to conclude as a treatment failure.

## Methods

### Selection of Patients

From July 1992 to December 2017, more than 19 000 patients were treated by GKRS in Marseille. In the study, patients with sporadic VS with an MRI follow-up of 3 years or more were included, and patients with neurofibromatosis or with a history of prior microsurgical resection or SRS before GKRS were excluded. In addition, patients who did not have appropriate and/or complete clinical and radiological assessment pre- and post-GKRS were excluded.

Since the beginning of our experience, a database has been created to register all clinical, radiological, and physical data corresponding to the treatments. The database was maintained prospectively over the follow-up.

The study was approved by the ethics committee of the Assistance Publique des Hôpitaux de Marseille (AP-HM) and by the French College of Neurosurgery (IRB00011687).

### Gamma Knife Treatment

Stereotactic radiosurgery was performed depending on the period with the Gamma Knife model B, C, 4C, Perfexion, or ICON (Elekta AB). After the placement of the Leksell G frame (Elekta AB) under local anesthesia, a stereotactic 1.5-T MRI scan was performed (sequences: 1-mm thick axial T1 high-resolution post-Gadolinium enhancement, constructive interference in steady state with and without enhancement, and Fat Sat). Additionally, a cranial CT scan (also 1-mm-thick axial slices) was performed to better visualize the inner bony ear structures and to check the absence of MRI distortion.^4,5^ Multiple-isocenter, conformal dose planning was performed using the Leksell KULA system and then using the GammaPlan software (Elekta AB) with the aid of the imaging set. The marginal dose was set to either 11 or 12 Gy, depending on the hearing status at GKRS as soon as 1992.

After GKRS, patients were routinely followed by clinical and radiological evaluation at 6 months, then 1, 2, 3, 5, 7, and 10 years post-GKRS. After 10 years the follow-up was proposed every 5 years. The patients were followed either in the GK department or by their referring practitioner (with results shared with the Marseille GK department) for the patients living far or abroad.

### Tumor Volume Measurements

All tumor volumes were measured on 1- to 1.5-mm-thick axial T1 post-Gadolinium enhancement (1.5 T MRI). Patients with insufficient quality of imaging were rejected from the study.

As the first treatments were performed with the KULA system, the tumor volume was calculated based on 5 measurements on the axial, coronal, and sagittal planes from the MRI films.^3^ With the integration of the GammaPlan software, those measurements became computer-assisted. Finally, with the inception of 3D sequences in routine MRI practice, segmental volumetry enabled a measurement of the tumor volume through manual delineation and automatic computation. The 5-axis volumetry was kept as a reference in routine,^6^ and then the corresponding data were available for all patients over the entire follow-up period. The measures were performed by 3 practitioners (J.R., C.D., and A.B.) and all were finally checked by one (A.B.).

### Treatment Failure

Treatment failure was defined as a need for a second treatment, either microsurgical resection or SRS, due to sustained tumor progression associated or not with clinical symptoms.

In our early experience, treatment failure was decided after a 3-year follow-up. Since then, we have reported that at 3 years post-GKRS, 22% of patients still have a tumor volume superior to the baseline^7^; defining tumor control as tumor volume at 3 years lower than baseline^8^ is not suited. Since then, our definition of treatment failure has evolved. If the tumor volume and the clinical condition are compatible, we propose a close follow-up with an MRI every year and tend to wait at least 5 years before concluding on the treatment failure. Thus, based on our updated strategy, some patients treated in the early period were prematurely proposed a second treatment. We retrospectively analyzed our patients classified as failure and defined if we would propose a second treatment or wait. These latter cases are classified in our study as “wait.” For the patients for whom we would have the same attitude, we classified them as “failure.”

### Evolution of the Tumor Volume

The objective was to group patients according to the dynamics of the evolution of the tumor volume (TumourVolume). The data available for each patient were, therefore, Xij measurements performed at certain Tij time moments, where i denoted the patient and j the MRI number. Thus, the screening MRI for a patient i would be Ti0. Note that these temporal moments can be very different from one patient to another, as well as the number of measurements. We were therefore left with data that were time series of different lengths, sampled at different times. In order to compare them and apply a clustering algorithm, the data were reprocessed as follows:

- For each patient i, the raw measurements were transformed into relative measurements, calculating the evolution of the tumor in relation to the time of screening, Rij = (Xij − Xi0)/Xi0 × 100.
- A priori moments of observation at 6, 12, 18, 24, . . . months were set.
- For each patient i, the Rij values at these times were determined by linear interpolation.

Depending on the observation period of each patient, some of these values (at the end of the trajectory) may be missing (censored data). Once the curves were aligned via linear interpolation, clustering was performed with a hierarchical method, and a dissimilarity was calculated from the correlation between the curves. Thus, the dissimilarity between patient i and patient iʹ is d(i, iʹ) = 1 − cor(Ri, Riʹ), with the correlation calculated on the 2 interpolated curves.

### Clustering

To define volumetric patterns representative of long-term trajectories rather than some initial pseudo-progression, we selected patients with an MRI follow-up of at least 3 years. The clustering was performed in 2 steps: Definition of the patterns of evolution based on a subset of patients with the most comprehensive follow-up and then the assignment of the remaining patients on a best-fit basis. Thus, the clustering was initially performed on the patients for whom the delay between the first MRIs was the shortest. The baseline data was then divided into 2 sets: patients with at least 4 MRIs (including screening one) in 3.5 years (6 months of time margin are left) were used to characterize the patterns. Patients with less than 4 MRIs in 3.5 years (including screening one) were used to test the patterns and a posteriori assigned to the best-fitting clusters. In order to reintroduce in the clusters the patients not initially selected (less than 4 MRIs during the first 3.5 years), the average of each cluster was calculated, and the correlation of their trajectories with the class averages was assessed. Each patient was finally assigned to the class with the maximum positive correlation.

This approach mimics a fairly classical approach of supervised learning, partitioning the starting data into a learning set and a validation set. In this case, where it is an unsupervised problem, it is a question of using the most complete data to learn the main patterns and then assigning to these patterns the more scattered and less complete data.

After having interpolated the trajectories of patients in terms of relative volume and alignment, the question of the minimum length of the follow-up was raised. Knowing that the observation times of each patient were different, we evaluated the best delay of follow-up necessary to be the most accurate. The consistency over time of the different clusters was calculated using the adjusted rand index (ARI) and normalized mutual information (NMI).

An analysis of the discriminant variables was finally performed for each pattern. A *P* value <.05 was considered significant.

### Early Assignment to a Cluster

The delay of follow-up required to assign a patient to a cluster depends on the pattern. To determine the delay required to predict the correct pattern, the assignment to classes was calculated using only the information available at 12 months, then 18 months, . . . The consistency between the prediction and the available information was then compared with the ARI and NMI criteria.

## Results

### Population of Patients

From our comprehensive institutional radiosurgical database, we identified 4024 patients who underwent GKRS for a VS between July 1992 and December 2017. We excluded 177 patients with neurofibromatosis and 360 who had prior treatment (microsurgical resection or previous SRS in another center). A total of 1880 patients with follow-up shorter than 36 months or with a qualitative follow-up only (status shared by a referring practitioner, but not the corresponding MRIs) were then excluded.

Finally, a total of 1607 patients (738 M/869 F; sex ratio 0.85) were included in the study. Median age at GKRS was 57 years old (mean: 56, range: 14–86 years), 799 patients (49.7%) were treated on the right side and 808 (50.3%) on the left side. The VS was discovered due to hearing deficit (46%), tinnitus (23%), vertigo (15%), gait disturbance (6%), or incidental finding (10%). Sudden deafness was experienced by 18% of patients before GKRS. The median delay between diagnosis and GKRS was 9 months (mean: 19; range: 0–268 months). The demographic details of the study patients are presented in Table 1.

**Table 1.** Description of the Selected Population, Radiosurgical Parameters, and Tumor Evolution Post-Gamma Knife Radiosurgery (GKRS)

Parameters	Population
Number of patients (M/F)	1607 (738/869)
Median age at GKRS in years (mean; range)	57 (56; 14–86)
Side (R/L)	799/808
Median delay before diagnosis in months (mean; range)	9 (19; 0–268)
Symptoms at diagnosis	
Hearing deficit	46%
Tinnitus	23%
Gait disturbance	6%
Vertigo	15%
Incidental finding	10%
Sudden deafness before GKRS	18%
Median tumor volume at GKRS in mm^3^ (mean; range)	543 (1093; 2–13 039)
Tumor size based on Koos classification	
I	22%
II	42%
III	25%
IV	11%
Tumor characteristics	
Solid with homogeneous enhancement	77%
Solid with heterogeneous enhancement	16%
Cystic	7%
Fundus invasion based on Ohata classification	
A	8%
B	56%
C	26%
D	9%
E	1%
Median marginal dose in Gy (mean; range)	12 (12; 8–18)
Median number of isocenters (mean; range)	8 (11; 1–50)
Median MRI follow-up in months (mean; range)	67 (81; 36–322)
The number of patients followed more than	
36 months	1607
48 months	1209
60 months	970
120 months	267
180 months	73
240 months	12
300 months	2
Median tumor volume at last follow-up in mm^3^ (mean; range)	282 (732; 1–12 980)
Loss of central enhancement post-GKRS	40.6%
Need for the second treatment	6.3%

Median tumor volume at GKRS was 543 mm^3^ (mean: 1093; range: 2–13 039 mm^3^) with 22% of patients having a tumor classified as Koos grade I, 42% as grade II, 25% as grade III, and 11% as grade IV; 93% of patients had a solid tumor with either homogeneous contrast enhancement in 77% or heterogeneous enhancement in 16% and 7% had cystic tumors. The median marginal dose was 12 Gy (mean: 12, range: 8–18 Gy). Median number of isocenters was 8 (mean: 11; range: 1–50). Considering the fundus invasion, 8% of the VS were classified as Ohata A, 56% as Ohata B, 26% as Ohata C, 9% as Ohata D, and 1% as Ohata E. The radiosurgical parameters are detailed in Table 1.

The overall median MRI follow-up was 67 months (mean: 81; range: 36–322 months) with 1607 patients followed for 36 months or more, 1209 for 48 months or more, 970 for 60 months or more, 267 for 120 months or more, 73 for 180 months or more, 12 for 240 or more, and 2 for 300 months or more. At the last follow-up median tumor volume was 282 mm^3^ (mean: 732, range: 1–12 980 mm^3^). A loss of central enhancement was observed during the follow-up in 652 cases (40.6%). Treatment failure was observed in 6.3% of patients, with 2.4% of patients treated with a microsurgical resection (1.6% between 3 and 5 years post-GKRS) and 3.9% undergoing a second radiosurgery (2.2% between 3 and 5 years post-GKRS). Based on our current criteria, 2.1% of the patients would not have been retreated that early but would have been proposed a close follow-up.

### Clustering and Stability of the Clusters

A set of 927 patients was used to define the clustering. The remaining 680 patients were used to test the model and a posteriori assigned to the best-fitting clusters.

We tested several time intervals to define the patterns, ranging from 3 to 10 years (with a margin of 6 months). For each selected observation period, a clustering in 5 patterns was defined using the distance induced by the correlation. The consistency of the clusters is presented in Supplementary Tables 1 and 2. We observed a high consistency (0.8) for the clustering at 5 years with the tumor volume at long-term (10 years) using both the ARI and NMI. In other words, the clustering is reasonably stable starting from 5 years. Based on these results, the clustering at 5 years seemed to correspond to the most effective delay of follow-up, considering its reliability compared to the longer-term follow-up and the relatively acceptable delay suitable for clinical practice.

**Figure 1.**
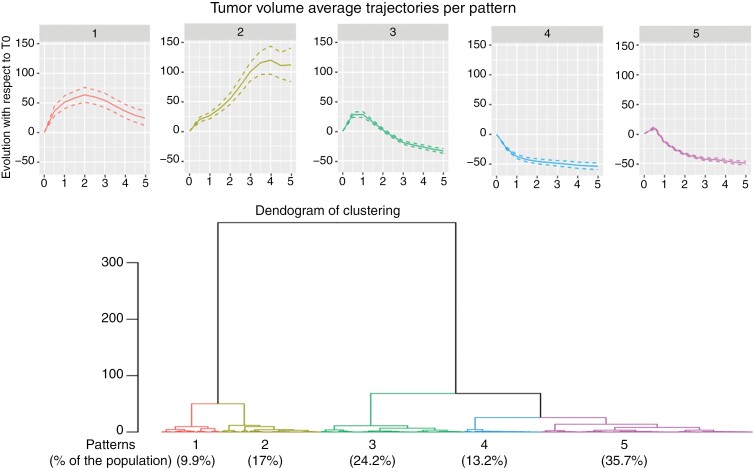
Dendogram of clustering representing the 5 patterns of volumetric evolution with a detailed trajectory for each pattern and standard deviation. Pattern 1 (red) includes 9.9% of the cohort, pattern 2 (yellow) 17.0%, pattern 3 (green) 24.2%, pattern 4 (blue) 13.2%, and pattern 5 (pink) 35.7%.

The 5 patterns using the clustering at 5 years (Figure 1) can then be described as the following evolution of tumor volume:

- Pattern 1: initial progressive increase during 2–3 years, followed by a slow regression; the delay to reduce under the baseline is longer than 5 years.
- Pattern 2: constant evolution—no change/constant stability or continuous increase.
- Pattern 3: initial rapid increase during the first year, followed by a plateau, then rapid regression.
- Pattern 4: constant regression since GKRS.
- Pattern 5: slight initial increase during the first months with a peak at 6 months–1 year, then rapid regression.

**Figure 2.**
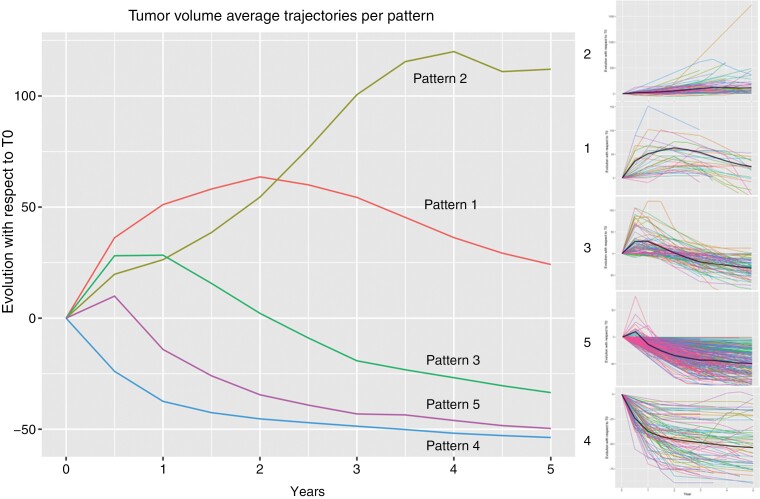
Tumor average trajectory per pattern is defined by the clustering (on the left). Individual tumor volume evolution for each pattern (on the right). These data include both patients used to define the patterns and those assigned a posteriori to a cluster. Patterns are represented in red (1), yellow (2), green (3), blue (4), and pink (5), respectively.

The distribution of the patients across the various patterns was as follows: 9.9% for pattern 1, 17.0% for pattern 2, 24.2% for pattern 3, 13.2% for pattern 4, and 35.7% for pattern 5. Global and individual data for each pattern are represented in Figure 2.

Further analyses were then performed on the clustering at 5 years.

### Discriminant Variables

The characteristics of each pattern are exemplified in Table 2.

**Table 2.** Characteristics of the Various Patterns of Vestibular Schwannomas Volumetric Changes and Discriminant Variables

	Pattern 1	Pattern 2	Pattern 3	Pattern 4	Pattern 5
Population	9.9%	17.0%	24.2%	13.2%	35.7%
Positive variables	Female sex Gait complaint Ohata B Short distance to fundus	Female sex Sudden deafness Tinnitus Younger age Short diagnosis delay	Long diagnosis delay Short distance to fundus Small number of isocenters	Ohata D Long distance to fundus Koos IV High number of isocenters	Male sex Long distance to fundus
Negative variables	Male sex Tinnitus Ohata C & D	Male sex Hearing complaint		Koos II	Female sex Vertigo complaint
Failure rate	2.2%	29.4%	0.5%	1.7%	0.3%

The positive discriminant variables for pattern 1 were female sex (*P* = .003), gait complaint (*P* = .013), Ohata B subtype (*P* < .001), and a short distance to fundus (*P* = .005). On the contrary, the negative discriminant variables were male sex (*P* = .003), tinnitus (*P* = .017), Ohata C (*P* = .029), and D subtypes (*P* = .004).

The positive discriminant variables for pattern 2 were female sex (*P* = .003), sudden deafness (*P* = .013), and tinnitus (*P* = .033). On the contrary, the negative discriminant variables were the male sex (*P* = .003), and hearing complaints (*P* = .001). In this pattern, the age at GKRS was significantly lower (*P* = .009) and the diagnosis delay before treatment was significantly shorter (*P* = .002).

For pattern 3, the diagnosis delay before treatment was significantly longer (*P* = .004), the distance to fundus shorter (*P* = .019), and the number of isocenters smaller (*P* = .004).

The positive discriminant variables for pattern 4 were Ohata D subtype (*P* = .003), Koos IV (*P* = .002), long distance to fundus (*P* = .004) and a high number of isocenters (*P* = .005). On the contrary, Koos II tumors were significantly underrepresented in this pattern (*P* = .004).

The positive discriminant variables for pattern 5, were the male sex (*P* < .001) and longer distance to the fundus (*P* = .003). On the contrary, the negative discriminant variables were female sex (*P* < .01) and vertigo complaint (*P* = .004).

### Probability of Failure

**Figure 3.**
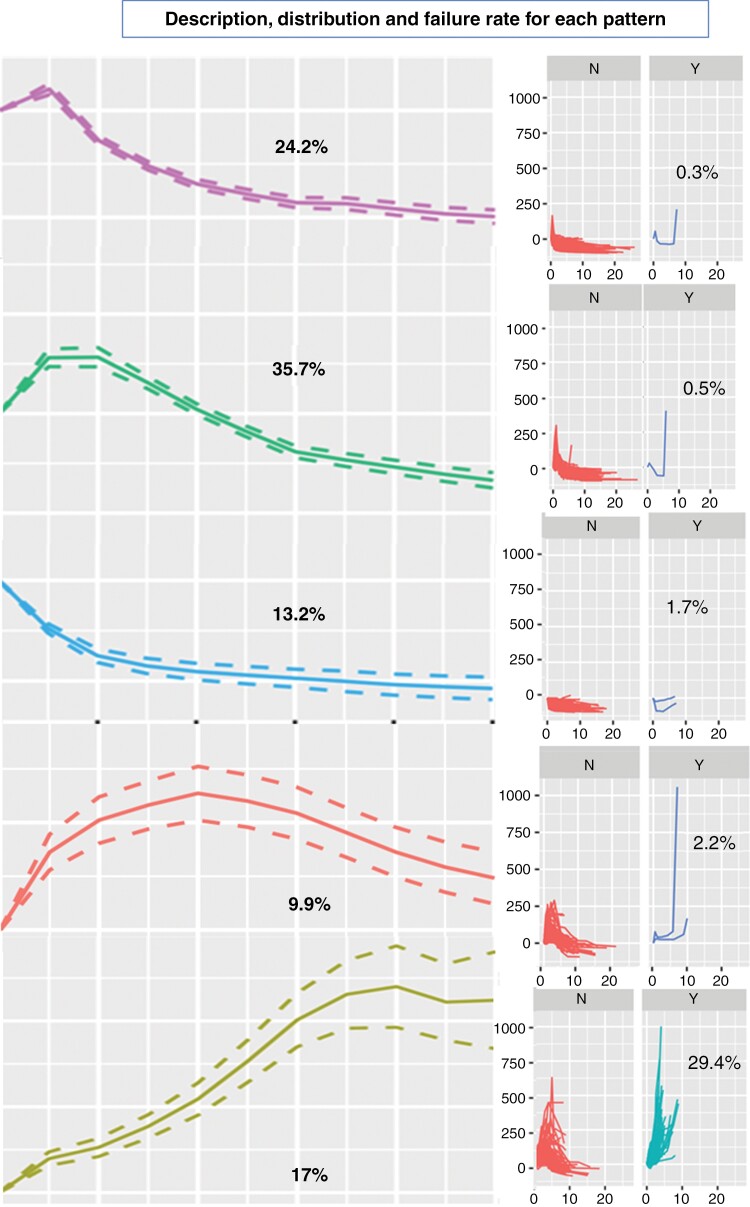
Description, distribution, and failure rate for each pattern with average trajectory on the left and individual trajectories on the right (red trajectories for controlled cases and blue for failures). The patterns are sorted based on failure rate (increasing from top to bottom). Patterns are represented in red (1), yellow (2), green (3), blue (4), and pink (5), respectively.

Almost all the patients with a diagnosis of failure or suspicion of failure (“wait” category) were clustered in pattern 2 (*P* < .001 and *P* < .001), with a probability of failure of 29.4% or suspicion of failure of 7.6%. The probability of having a failure for each pattern is represented in Figure 3.

### Delay Necessary to Predict the Evolution

A minimum of 3 years is required to assign the evolution of the tumor volume to the correct cluster with a high consistency (Table 3). Still, depending on the pattern, this delay varies from 24 (patterns 2, 3, and 5) to 36 months (patterns 1 and 4). Using ARI and NMI criteria, the consistency at 3 years reached 0.81 and 0.80, respectively.

**Table 3.** Probability of Correct Prediction Per Pattern of Vestibular Schwannomas Volumetric Changes Depending on the Duration of Follow-up

Probability of predicting the correct pattern	12 months	24 months	36 months	48 months
Pattern 1	22.5%	20.3%	73.5%	86.9%
Pattern 2	41.0%	71.0%	90.8%	94.7%
Pattern 3	49.2%	79.5%	88.1%	87.9%
Pattern 4	47.9%	61.5%	78.9%	77.0%
Pattern 5	88.6%	83.6%	92.9%	94.3%

## Discussion

### Early Characterization of Tumor Volume Dynamics

The goal of SRS is to control the tumor, and then prevent patients from potential direct consequences of tumor growth or complications of surgical resection. Thus, patients after SRS are waiting for months, if not years, before getting the conclusion of tumor control or failure. In the early days of SRS, it was suggested that VS controlled at 3 years post-SRS would not grow again.^8^ Based on these conclusions, it was decided to define the outcome of SRS at a 3-year follow-up.

In the early 2000s, Delsanti et al.^3^ proposed several patterns of volumetric and morphological changes of VS for tumor control: (1) pseudo-progression followed by progressive tumor shrinkage, (2) direct tumor shrinkage (no transient expansion), and (3) tumor stabilization; they were followed by several other authors.^9–11^ The group experiencing pseudo-progression can be divided into subgroups based on the definition of Pollock^9^: type 1: pattern characterized by initial volume increase followed by later tumor reduction to either the same or reduced size compared to radiosurgery; type 2: pattern characterized by tumor volume increase that persists, but the tumor does not enlarge on later imaging; type 3: pattern characterized by progressive tumor volume increase on serial imaging. In our study, these patterns have also been observed with our pattern 1 corresponding to the type 2 of Pollock. We also described, in this study, 2 patterns with transient expansion (patterns 3 and 5) differentiated by the importance and delay of the pseudo-progression.

### Pseudo-progression

The phenomenon of pseudo-progression, also called transient swelling or transient tumor enlargement, must be well known in order not to systematically classify these responses as treatment failures. In the early series, pseudo-progression was reported in only 5% of VS treated with radiosurgery.^12^ In more recent series, an incidence of 17% to 62% has been reported,^3,10,13^ but the definition was not always clearly stated.^14^ This phenomenon is often transient, tends to start 6 to 9 months after SRS, and resolves in the first 36 to 48 months.^10,13,15^ Nagano et al.^10^ found pseudo-progression after SRS in 77% of cases. This expansion resolved in 50% of cases at 18 months, and 90% of cases at 5 years. Breshears et al.^15^ reported that while it can take as long as 6.9 years for 90% of tumors with pseudo-progression to shrink back to treatment volume, 90% had peaked in size by 3.5 years following SRS. Additionally, 90% of tumors with pseudo-progression had begun enlarging by 3.2 years. While it may take close to a decade for controlled tumors to return to treatment sizes, they showed that most tumors with transient rather than persistent growth should decrease in size by approximately 4 years. Those with persistent enlargement beyond these points are increasingly likely to result in treatment failure.

Pseudo-progression in VS seems independent of age, gender, laterality, previous surgery, tumor volume, peripheral dose, conformity index, and dose rate^16,17^ and is in the vast majority of cases of no or little relevance to the patient.^13^ Controversy exists in the literature on how much tolerance a clinician should have for tumor enlargement following SRS.^15^ In the study by Pollock et al., the median volume increase was 75%, with a maximum of 280%.^18^ The same results were reported by our team.^7^ The amount of tumor enlargement might then not be an argument for tumor progression versus pseudo-progression. Still, if we compare this subgroup with patients considered as failure, although the mean initial growth at 6 months was similar to the group with pseudo-progression, the VSs in the failure subgroup grew more rapidly.^7^ As pseudo-progression is one of the major arguments against SRS for large VSs, it would be extremely beneficial if this effect could be predicted a priori. We reported in a previous study that VS with Koos grade IV had a tumor control rate almost as high as the small to medium VS.^19^ We have shown in this study that those tumors were more frequently classified in pattern 4 (constant tumor regression). This argument pleads in favor of SRS for the smallest Koos grade IV tumors, when clinically relevant.

Although in most cases this pseudo-progression results in a final volume smaller than baseline, a small percentage of patients will eventually show a tumor progression, then stabilization or late regression (after 5 years).^18,20^ Close and long-term follow-up is recommended in these cases. These patients raise the question of a treatment failure; the characterization and prediction of long-term evolution remains a challenge. In our study, these patients were mostly clustered in pattern 1, where the patients undergoing a retreatment represent only 2.2%.

### Predictive Factors of Tumor Control

No consensus is available to standardize the definition of responders and nonresponders for VS management.^21^ This may, in part, explain the wide range of treatment failure versus success rates in the literature, depending on the criteria adopted. The evident failure of tumor control after SRS, defined as sustained tumor growth, at least after 5 years, is rare (accounting for less than 5%). Factors like pretreatment tumor growth, tumor characteristics, and biologically effective dose (BED) have been studied, but none of these parameters demonstrated a clear impact on tumor control. Since the 1960s, marginal dose rates have decreased from 25 Gy down and are now internationally standardized to 11 to 13 Gy. Although this reduction in treatment dose did not seem to impact tumor control, other treatment parameters might influence the rate of failure or the dynamics of tumor evolution.

GKRS treatment times vary, even for the same prescription dose, due to variations in the collimator size, the number of isocenters, the dose rate depending on the decreasing activity of the cobalt-60 (^60^Co) sources, and the time gap between each of these exposures. The BED, a concept initially used in fractionated radiotherapy, incorporates both the prescribed dose and treatment time.^22^ Tuleasca et al. recently showed that higher BED linearly correlated with tumor volume changes.^23^ On the contrary, Villafuerte et al. reported in a retrospective study that BED was not associated with tumor control.^24^ Although these findings are still controversial,^25^ BED calculations may indicate the importance of reporting overall time to reflect the biological effectiveness of the total physical dose applied.^26^ The impact of the BED on the patterns will surely have to be evaluated, all the more with the rise of software like Lightning inverse planning.

The impact of pretreatment growth has been studied by various authors. Delsanti et al. reported more important pseudo-progression for patients with a high rate of tumor growth before SRS.^3^ More recently, Chang et al.^27^ showed no significant differences in pretreatment growth rates between tumors having a transient post-SRS increase and those who had not. Yet the series was small and the median follow-up short. The same results were reported by Larjani et al.^28^ and Timmer et al.^29^ On the contrary, Marston et al.^30^ reported in patients with clear growth before SRS more short-term growths after SRS, which were interpreted as failures. However, it was pointed out that the short follow-up prevented from distinguishing actual progression from pseudo-progression.^31^ In this study, we have not evaluated the impact of pretreatment growth.

It has been suggested that each histological component may have a specific response to SRS. Homogeneous tumor enhancement on MRI has been reported to be associated with a risk of insufficient tumor shrinkage.^20^ Pendl et al. reported in 1996 a higher rate of early failure in cystic VS, leading some authors to consider cystic VS as a contra-indication for SRS.^32^ In our early experience, we also considered that cystic VSs were more at risk of failure.^33^ Yet, at that time the number of patients treated for cystic VS was small. Since then, some authors, based on larger series with longer follow-ups, reported that the tumor control rate of cystic VS was equal^34^ or even higher^35,36,37^ than that of solid VS after GKRS, with a much shorter mean tumor half-reduction time than solid VS.^37^ In our large series, the tumor type (cystic or not) was not a prognostic factor of failure and was not characteristic of a particular pattern.

### New MRI Sequences

With the development of new sequences like perfusion or diffusion-weighted imaging, the promise was also to bring new information in tumoral diagnosis, grading, or prognosis. The correlation between the evolution of ADC and treatment response has been discussed. Yet, this correlation is still controversial.^35,21^ Like us, several authors also proposed to categorize the patients according to the radiological evolution over time. However, prospectively assigning a patient to one of these categories is still difficult. New MRI sequences or post-processing algorithms, such as arterial spin labeling, multinuclear MRI,^38^ dynamic contrast-enhanced MRI,^39^ and diffusion coefficient mapping or radiomics,^40^ could have a potential interest in differentiating tumor growth from the effects of radiation.^41^ Thus, Langenhuizen et al. and Zanetti et al.^40,42^ have shown the feasibility of predicting the long-term SRS treatment response of VS on an individual basis, using MRI-based tumor texture features with machine learning systems. Although radiomics may predict VS response to radiosurgery, avoiding long-term follow-up, these techniques are not routinely used in clinical practice and will have to be evaluated.^40,42^

### Volumetry

Microsurgical resection^43^ and/or additional SRS^44^ are the 2 options in case of treatment failure, the decision being based on the tumor size, clinical tolerance, and habits of the managing team. Exposing patients to unnecessary second treatment is discouraged. Published series of VS resections after “failed” radiosurgery illustrate the practice of early surgery after SRS (before 3 years).^45,46^ To mitigate such superfluous treatments, a consensus on the definition of treatment failure has to be obtained; accurate measurement of tumor volume is then required. 3D volumetry was proven to be more sensitive than linear-dimension measurements in detecting changes.^47^ Although segmental volumetry is deemed to be the gold standard, on the recent EANO guidelines, linear measurements are still used to assess tumor growth,^48^ showing that no clear consensus has yet been reached. In this study, we kept the 5-axis volumetry to ensure consistency of measures over the entire period under analysis.^6^

### Pace of Follow-up

The aim of SRS for VS is tumor control, contrary to surgery where the tumor is resected. The course of VS after SRS is not uniform^7^; the proper way to describe these changes is to obtain a sequential iterative MRI scan. The amount of post-SRS MRIs necessary to decide on the pattern of evolution raises the question of cost-effectiveness. Some authors have proposed to omit routine MRI before 12 months in clinically stable patients.^49^ Based on this study and our experience, we believe that the closer the first MRIs, the more accurately the tumor evolution will be predicted.

### Minimum Follow-up

In the early 1990s were considered controlled VSs smaller at 3 years than baseline.^8^ The demonstration of late pseudo-progression has obsoleted this statement.^3,18^ Mindermann et al. suggested that VSs that begin to enlarge after 24 to 36 months usually correspond to treatment failure.^13^ Yet, cases of delayed or sustained pseudo-progression then regression have been published.^3,31,41^ Short follow-up is an important limitation in many studies, as VSs can have transient growth even after 5 years post-SRS, which can take several years to resolve. We have shown that the clustering at 5 years was the most reproducible; the minimal delay (in the absence of major clinical symptoms) required to predict a failure should be 5 years. Yet, this rule can be adapted in case of symptomatic worsening.

### Limits of the Study

This study holds several limitations. First, to maximize the cohort and follow-up, we included patients treated in the 1990s. At that time, 3D volumetry was not an option. The volumes were then estimated based on the 5-axis volumetry, which might impact the results. Yet, we have shown that this method is reliable^6^ and all the measures have been done using this method. Second, in our cohort, patients have been treated with a median marginal dose of 12 Gy. As some centers around the world are using a slightly higher regimen of dose (13 Gy), it is a matter of debate whether those doses and plans could have slightly different dynamic responses. The same questioning can be applied to dose distribution variations (eg, mean dose and/or central dose), especially in the context of significantly lower integrated dose in the target volume with the use of Lightning inverse planning.^50^ Third, some patients did not follow the recommendations for the MRI follow-up. Furthermore, the delay between the planned MRIs increases with the length of follow-up (3, 5, 7, 10, and then 15 years post-GKRS), reducing the amount of data available in the long term. These missing MRIs can lead to inaccuracy in the linear interpolation model when the delay between 2 MRIs is too long. To reduce this bias, we selected for the clustering only the patients with a comprehensive follow-up in the first years post-SRS. Fourth, some patients were excluded due to short follow-up, insufficient quality of the imaging, or qualitative follow-up only. This may lead to a selection bias. Finally, because this study focused on radiological data, we did not consider the clinical follow-up; in case of symptomatic progression, the decision of a second treatment should be based primarily on the clinical tolerance of the patient.

## Conclusions

The definition of failure in terms of delay of follow-up and dynamics of evolution of the tumor volume is still a matter of debate. We defined 5 patterns, with one pattern gathering almost all cases of treatment failure. Due to the high variability of individual profiles of volumetric evolution after SRS, the definition of these 5 robust distinct patterns is likely to help the physicians tremendously to distinguish successful tumor control from potential failure in the longer term. Although a delay of 3 years can predict the evolution in up to 80%, we would advocate for no retreatment decision before 5 years post-GKRS, with the exception of a clinical deterioration requiring surgical decompression. To decide if the dynamics of evolution can be predicted either at GKRS or in the early follow-up on an individual basis, further investigations are required.

## Supplementary material

Supplementary material is available online at *Neuro-Oncology* (https://academic.oup.com/neuro-oncology).

noae187_suppl_Supplementary_Table_S1

noae187_suppl_Supplementary_Table_S2

noae187_suppl_Supplementary_Material

## Data Availability

The data used for the study are available upon request from the corresponding author.
