# Supplementary material for: Dynamics of tumor evolution after Gamma Knife radiosurgery for sporadic vestibular schwannoma: Defining volumetric patterns characterizing individual trajectory
Source: Neuro Oncol. 2024 Sep 16;27(2):545–56. doi: 10.1093/neuonc/noae187 (PMC11812029; doi:10.1093/neuonc/noae187)
Supplement: noae187_suppl_Supplementary_Table_S1 [file noae187_suppl_supplementary_table_s1.docx]

|  | **Cluster at 3 years** | **Cluster at 4 years** | **Cluster at 5 years** | **Cluster at 6 years** | **Cluster at 7 years** | **Cluster at 8 years** | **Cluster at 9 years** | **Cluster at 10 years** |
| --- | --- | --- | --- | --- | --- | --- | --- | --- |
| **Cluster at 3 years** | 1.00 | 0.45 | 0.48 | 0.40 | 0.42 | 0.37 | 0.44 | 0.48 |
| **Cluster at 4 years** | 0.45 | 1.00 | 0.47 | 0.46 | 0.51 | 0.34 | 0.43 | 0.43 |
| **Cluster at 5 years** | 0.48 | 0.47 | 1.00 | 0.67 | 0.65 | 0.58 | 0.77 | 0.80 |
| **Cluster at 6 years** | 0.40 | 0.46 | 0.67 | 1.00 | 0.62 | 0.49 | 0.55 | 0.60 |
| **Cluster at 7 years** | 0.42 | 0.51 | 0.65 | 0.62 | 1.00 | 0.49 | 0.69 | 0.68 |
| **Cluster at 8 years** | 0.37 | 0.34 | 0.58 | 0.49 | 0.49 | 1.00 | 0.57 | 0.60 |
| **Cluster at 9 years** | 0.44 | 0.43 | 0.77 | 0.55 | 0.69 | 0.57 | 1.00 | 0.93 |
| **Cluster at 10 years** | 0.48 | 0.43 | 0.80 | 0.60 | 0.68 | 0.60 | 0.93 | 1 |

**Table°1:** Consistency of the different clusters based on ARI
