# Supplementary material for: Dynamics of tumor evolution after Gamma Knife radiosurgery for sporadic vestibular schwannoma: Defining volumetric patterns characterizing individual trajectory
Source: Neuro Oncol. 2024 Sep 16;27(2):545–56. doi: 10.1093/neuonc/noae187 (PMC11812029; doi:10.1093/neuonc/noae187)
Supplement: noae187_suppl_Supplementary_Table_S2 [file noae187_suppl_supplementary_table_s2.docx]

|  | **Cluster at 3 years** | **Cluster at 4 years** | **Cluster at 5 years** | **Cluster at 6 years** | **Cluster at 7 years** | **Cluster at 8 years** | **Cluster at 9 years** | **Cluster at 10 years** |
| --- | --- | --- | --- | --- | --- | --- | --- | --- |
| **Cluster at 3 years** | 1.00 | 0.56 | 0.56 | 0.53 | 0.54 | 0.51 | 0.54 | 0.56 |
| **Cluster at 4 years** | 0.56 | 1.00 | 0.62 | 0.61 | 0.64 | 0.51 | 0.57 | 0.57 |
| **Cluster at 5 years** | 0.56 | 0.62 | 1.00 | 0.73 | 0.76 | 0.67 | 0.78 | 0.80 |
| **Cluster at 6 years** | 0.53 | 0.61 | 0.73 | 1.00 | 0.68 | 0.65 | 0.64 | 0.67 |
| **Cluster at 7 years** | 0.54 | 0.64 | 0.76 | 0.68 | 1.00 | 0.65 | 0.75 | 0.75 |
| **Cluster at 8 years** | 0.51 | 0.51 | 0.67 | 0.65 | 0.65 | 1.00 | 0.70 | 0.73 |
| **Cluster at 9 years** | 0.54 | 0.57 | 0.78 | 0.64 | 0.75 | 0.70 | 1.00 | 0.93 |
| **Cluster at 10 years** | 0.56 | 0.57 | 0.80 | 0.67 | 0.75 | 0.73 | 0.93 | 1 |

**Table °2:** Consistency of the different clusters based on NMI.
