# Supplementary material for: Dynamics of tumor evolution after Gamma Knife radiosurgery for sporadic vestibular schwannoma: Defining volumetric patterns characterizing individual trajectory
Source: Neuro Oncol. 2024 Sep 16;27(2):545–56. doi: 10.1093/neuonc/noae187 (PMC11812029; doi:10.1093/neuonc/noae187)
Supplement: noae187_suppl_Supplementary_Material [file noae187_suppl_supplementary_material.docx]

***Supplementary material***

**Table°1:** Consistency of the different clusters based on ARI.

**Table °2:** Consistency of the different clusters based on NMI.
